# Transmission Attributes of Periurban Malaria in Lusaka, Zambia, Precedent to the Integrated Vector Management Strategy: An Entomological Input

**DOI:** 10.1155/2012/873852

**Published:** 2012-09-30

**Authors:** Emmanuel Chanda, Kumar S. Baboo, Cecilia J. Shinondo

**Affiliations:** ^1^National Malaria Control Centre, Ministry of Health, Directorate of Public Health and Research, Chainama College Grounds, P.O. Box 32509, 10101 Lusaka, Zambia; ^2^Department of Community Medicine, School of Medicine, University of Zambia, P.O. Box 50110, 10101 Lusaka, Zambia; ^3^Department of Biomedical Sciences, School of Medicine, University of Zambia, P.O. Box 50110, 10101 Lusaka, Zambia

## Abstract

Globalization and urbanization with their inherent developmental activities and ecological transformations impact on malaria epidemiology. Entomological factors involved in malaria transmission in periurban Lusaka were assessed prior to vector control reintroduction. Data was collected through standard entomological and epidemiological protocols and a pretested structured questionnaire. Larval habitats were characterized as transient (43%), semipermanent (36%), and permanent (21%). *Anopheles arabiensis* and *An. gambiae ss.* were the only vectors identified. A shift in vector population was noted, with the later outnumbering the former. *Plasmodium falciparum* monoinfection rates were 25.6% (95% CI: 20.9–30.7) (*n* = 297). Parasitaemia was 31.8% (95% CI: 23.2–42.2), 25.7% (95% CI: 13.5–41.3), and 23.3% (95% CI: 17.4–29.6) in under 5, 5 to 14, and above 15 age groups, respectively. Low knowledge levels on vector control tools with an average of 7 residents per household were also observed. This study confirmed a local malaria transmission paradigm. The epidemiology necessitated deployment of an integrated vector management strategy with intensified information education and communication.

## 1. Introduction

Malaria remains a serious global health problem, killing more than one million people per year. The global community has recently had many successes in malaria control. The number of malaria cases has fallen by more than 50% in 43 countries over the past decade [1]. A modeling analysis of malaria prevention activities in 34 African countries suggested that about 730,000 lives were saved between 2000 and 2010, with nearly three quarters of those since 2006 [2]. Funding commitments for malaria have increased nearly 15-fold, from approximately US$ 100 million in 2003 to nearly US$ 1.6 billion in 2010; interest and commitment at global and country levels are very high [3]. However, the problem of malaria parasite transmission remains enormously grave in sub-Saharan Africa where at least 85 to 90% of deaths are attributable to the disease [4–7]. 

Malaria transmission is driven by a complex interaction of the vector, parasite, human host, and the environment, and is governed by different ecological and social determinants [8, 9]. Globalization and urbanization with their inherent developmental activities and associated ecological transformations have a significant impact on malaria epidemiology [10, 11] and have invariably exacerbated the situation. Malaria transmission depends markedly on local environmental conditions and other compounding factors, that is, presence of drug-resistant parasites and insecticide resistant vectors [12, 13], environmental changes [14], economically driven human population increase and migration [15], poverty levels, climatic changes, natural disasters and political upheavals [16], adaptability of malaria vectors to changing environments [17, 18] and limited investment in research, drug discovery, and optimisation of malaria vector control programmes.

Transmission patterns and severity of malaria are influenced by the geographic attributes and the socioeconomic environment that vary significantly by city, season, and age group [19]. Accordingly, entomological profiles and clinical patterns are known to vary between urban, periurban, and rural environments [20]. Well-developed urban areas are mostly fringed by underdeveloped and inadequately serviced periurban areas experiencing the highest population growth rates [21] and often lacking infrastructure.

Malaria transmission in peri-urban areas is mostly ascribed to increased vector breeding created by the agricultural and construction activities, lack of drainage of surface water [18, 22, 23], human vector contact due to poor housing and overcrowding [11], and low immunity in children under five and pregnant women, thus increasing the risk of severe disease [19]. In Zambia, between 1950 and early 1980s, vector control reduced malaria cases to a notifiable disease in most urban areas [24]. Ngandu and coworkers reported the resurgence of malaria cases in urban and peri-urban Lusaka [25]. *In vivo* sensitivity tests were also conducted with *Plasmodium falciparum* patients in Lusaka [26], but whether these infections were acquired in urban Lusaka itself or in rural areas was not clear.

Owing to malaria cases resurgence and paucity of entomological data, specific local investigations to appraise and confirm malaria transmission in peri-urban Lusaka were required before approaches to malaria vector control could be considered. We report here on malaria vectors, parasite prevalence rates in febrile patients and knowledge and attitudes of the community pertaining to malaria, precedent to the implementation of the integrated vector management (IVM) strategy.

## 2. Materials and Methods

### 2.1. Study Site

**Figure 1:**
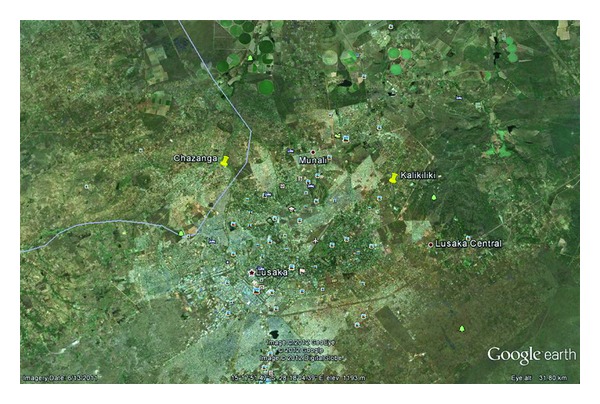
Map of greater Lusaka showing the periurban study site locations.

Zambia is a landlocked country in southern Africa with an estimated population of 13 million people, 45% are children below 15 years of age [27]. Malaria is endemic across the entire country with transmission peaks coinciding with the rainy season from November to April. This study was conducted in two spatially segregated and randomly selected peri-urban locations of Lusaka district; Chazanga and Kalikiliki (Figure 1) during the cold-dry season from May to July 2003. The two sites have similar ecological characteristics and stretch out in an epidemiological zone characterized by low malaria transmission.

### 2.2. Mosquito Collections and Laboratory Processing

Mosquito larvae were collected from breeding sites using WHO-standard 250 mL dippers [28], transported to the insectary at the National Malaria Control Centre in Lusaka, and reared to adults while being fed on 1 part yeast and 2 parts dog biscuit. Adults were maintained on 10% sugar solution at 25 ± 2° centigrade temperature and 70–80% relative humidity. 

Mosquito breeding sites were characterized into three different categories: transient, semipermanent, and permanent. Transient breeding site refers to temporal water collections, semi-permanent ones are those that would persist for a considerable period of time. Permanent breeding site refers to water bodies available throughout the year.

Adult mosquitoes were collected by the pyrethrum spray catch (PSC) between 06:00 hrs and 08:00 hrs in randomly selected households [28]. *Anopheles* mosquitoes were identified morphologically using standard keys for anophelines of southern Africa [29, 30] and to species by the polymerase chain reaction (PCR) molecular method of Scott et al. [31]. 

### 2.3. Parasitemia in Febrile Patients

*Plasmodium falciparum* infection was determined among febrile patients at health facilities in the study sites. Blood from randomly selected subjects who presented to the health center with febrile symptoms and consenting to participate was screened for parasite species and gametocytes by microscopy using 4% Giemsa thick and thin blood smears for 30 minutes [32]. The age range of subjects was stratified into three age categories: 6 months <5, 5 to <15, and 15 years and over. Participants with positive slide tests were offered free treatment with artemisinin-based combination therapy (ACT) according to Zambia national malaria control programme treatment policy guidelines [33].

### 2.4. Knowledge and Attitudes

A pretested structured questionnaire was administered to 150 randomly selected respondents, tested for malaria, to determine community knowledge and attitudes as regards malaria, family demographic data, and possibility of malaria importation from rural areas. 

### 2.5. Data Management and Statistical Analysis

Randomization was calculated for both study sites. Data was collected and entered in Excel spread sheets (Microsoft Corporation) and statistically analyzed by employing Epi Info version 3.2.2. The Chi-square (*χ* ^2^) test was used to determine the differences in parasite prevalence between age categories.

### 2.6. Ethical Consideration

Ethical approval for the research was granted by the University of Zambia Research Ethics Committee (Assurance number. FWA00000338 IRB00001131 of IOR G0000774). A freely administered informed consent was given to respondents and householders for participation in the study.

## 3. Results

### 3.1. Mosquito Collections

**Figure 2:**
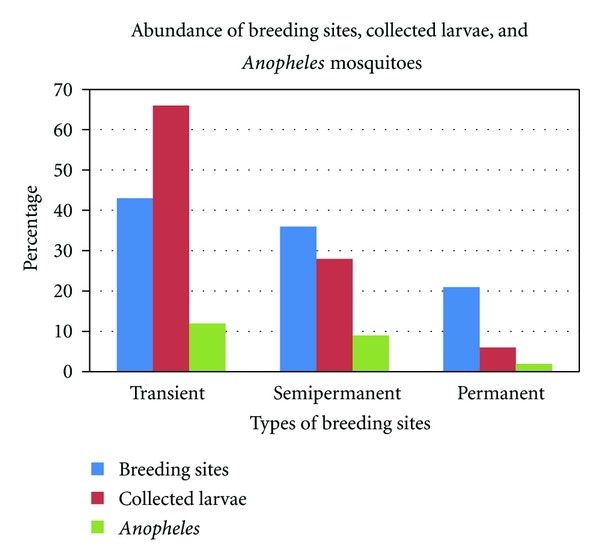
Abundance of breeding sites, collected larvae, and *Anopheles* mosquitoes.

Of 1840 larvae collected, 66% (95% CI: 65.7–68.1) were from transient (gardens and abandoned building foundations), 28% (95% CI: 25.6–29.6) semipermanent (abandoned shallow wells and ditches that followed in the wake excavations for building sand or quarrying) and 6% (95% CI: 5.4–7.7) permanent water bodies (perennial streams and dams) (Figure 2). Anophelines accounted for only 21.9% (95% CI: 20.1–23.9). The density of *Anopheles* larvae was comparatively higher in semipermanent (31.7%) followed by the permanent (25%) and transient habitats (17.5%) (Figure 2 and Table 1).

**Figure 3:**
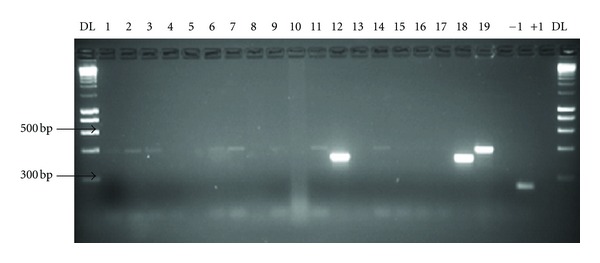
DNA bands produced by ribosomal DNA-polymerase chain reaction (PCR) amplification from the different species in the *Anopheles gambiae* complex from Chazanga. DL: 1-kp DNA ladder size standards, +1: positive control (*A. arabiensis*), −1: negative control. The sample DNA in each of the lanes was as follows: 1, 2, 3, 6, 7, 11, and 14 were amplified for *A. gambiae* ss. (390 bp). 12 was amplified for *A. arabiensis * (315 bp).

**Figure 4:**
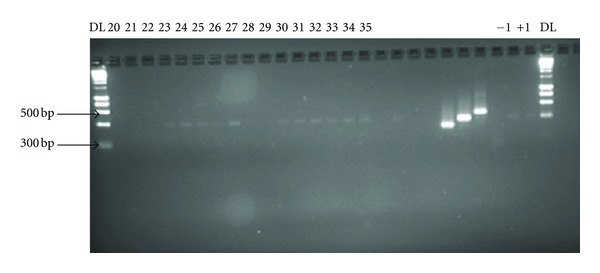
DNA bands produced by ribosomal DNA-polymerase chain reaction (PCR) amplification from the different species in the *Anopheles gambiae* complex from Kalikiliki. DL: 1-kbp DNA ladder size standards, +1: positive control (*A. arabiensis*), and −1: negative control. The sample DNA in each of the lanes was as follows: 23, 24, 25, 26, 27, 30, 31, 32, 33, 34, and 35 were amplified for *A. gambiae *ss. (390 bp).

Anophelines constituted 12.83% (95% CI: 8.7–17.9) of the 203 adult mosquitoes collected (Table 1). The mosquito male-to-female ratios and densities per room was 0.59 to 0.26 and 1.7 to 15 for *Anopheles* and *Culex,* respectively. A total of 30 *An. gambiae *ss. were subjected to molecular assays including those reared from larvae. 11 could not amplify a PCR product. All specimens from Kalikiliki (*n* = 11) and Chazanga (*n* = 7), amplified for *An. gambiae *ss. and only 1 from Chazanga amplified for *An. arabiensis* (Figures 3 and 4).

### 3.2. Parasitemia in Febrile Patients

A total of 297 randomly selected febrile patients were recruited into the study (Table 2). The age of the subjects ranged from 6 months to 60+ years. Seventy-six (25.6%) were positive for malaria parasites with 100% *Plasmodium falciparum* parasite monoinfection. Among the positive slides, 75 (98.7%) exhibited ring form trophozoites and only 1 (1.3%) showed gametocytaemia. The parasitemia in febrile patients per age group was 31.8% (95% CI: 23.2–42.2) for the 0–4 years group, 25.7% (95% CI: 13.5–41.3) for 5 to 15 years, and 23.3% (95% CI: 17.4–29.6) for the 15 years and over (*P* > 0.05).

### 3.3. Knowledge and Attitudes

Of the 150 respondents 18% (95% CI: 12.4–24.6) were male and 82% (95% CI: 75.4–87.3) were female. The mean age was 29.9 with a range of 18 to 53 years. Forty-eight percent exhibited good knowledge of malaria as a disease. Sixty-three percent were knowledgeable about malaria transmission. Seventy-nine per cent were conversant with causes, signs and symptoms. Sixty-two per cent showed awareness of what to do when they suspected malaria and only forty-six per cent were knowledgeable about vector control interventions. Family demographic data showed an average of seven residents with at least one child under five years per household. Eighty-one per cent of respondents had no history of travel outside Lusaka. There was positive association between knowledge and malaria prevalence in peri-urban Lusaka (*P* < 0.05).

## 4. Discussion

The malaria vectorial system in Zambia comprises of *An. gambiae *ss.*, An. arabiensis,* and *An. funestus* [34, 35], with great divergence in their malaria transmission potential, spatial segregation, and temporal heterogeneity [36, 37]. The pioneering malaria control efforts in the country [38, 39] stimulated unprecedented enthuse in entomological studies [36, 40–44]. Recent studies have demonstrated the presence of *An. nili*, *An. funestus-*like, and *An. rivulorum *although their role in malaria transmission in Zambia is yet to be established [45]. 

Urban areas are perceived not to support significant levels of malaria transmission [18]. In this study, three kinds of mosquito breeding habitats: transient, semipermanent, and permanent were characterized with appreciable spatial heterogeneity (Figure 2). *An. gambiae* is known to exploit small open temporal habitats with less predation, increased warmth, and more algae [46]. However, more *Anopheles* larvae were collected from semipermanent habitats than from permanent and transient habitats (Figure 2 and Table 1). This could explain the role of urban development related activities in supporting high malaria transmission levels as observed in peri-urban Lusaka. 

While formal urban development typically reduces mosquito densities, informal urbanization has been shown to alter the vector species composition within the *An. gambiae* complex in sub-Saharan Africa, [47]. To illustrate, earlier studies conducted in Zambia indicated 100% *An. arabiensis* [35, 36]. Nevertheless, the profound demographic and extensive environmental changes that have followed in the wake of urbanization have changed the stratification of the vectors. This study demonstrates coexistence of *An. gambiae* ss. and *An. arabiensis* with the former greatly outnumbering the later in complete absence of *An. funestus*. Notably, the predominance of *An. gambiae *ss. validates the premise that informal urban development does transform vector species composition.

The presence of *An. arabiensis*, a species that is typically difficulty to control by IRS and ITNs, and the predominance of the *An. gambiae* ss. which is characteristically amenable to control by IRS and ITNs [48] could have implications for the malaria control programme. The sympatric-existence of these vectors demonstrates the need for an integrated approach for malaria vector control. This study was characterized by low number of mosquito collections due to the unfavorable prevailing environmental conditions during the cold season, lack of data on chromosomal forms of *An. gambiae* ss. and transmission determining parameters, that is, vector infectivity. However, early entomological work in Zambia reported a sporozoite rate of 1.4% in *An. arabiensis *in Lusaka [44]. Notably, there is still a clear paucity of data on malaria vector bionomics in the country. 

Malaria had been known to be hyperendemic in hot riverine valleys with perennial transmission, meso-to hypoendemic on plateaus, and hypo-endemic in urban areas of Zambia [49]. Between 1969 and 2000, parasite rates ranged from 2.0 to 26.4% across the country [39], with parasite species of 86.8% *P. falciparum *and 13.2% *P. malariae *in Ndola rural [50]. By 1999, parasite species was over 97% *P. falciparum *[49]. These findings are corroborated in this study with 25.3% parasitaemia among febrile patients with 100% *P. falciparum* monoinfections. This upsurge of frequency of febrile malaria was further aggravated by the development of chloroquine resistance [51]. Deployment of effective control tools has transformed the epidemiological profile from countrywide high endemicity to three distinct epidemiological strata: very low transmission and parasite prevalence of <1%, low transmission (<10%), and persistent high transmission (>20%).

The prevalence rate of malaria in children under five years is dependent on the intensity of transmission and declines with age as immunity develops and is thus a good indicator of a recent transmission of malaria [52]. The highest prevalence of malaria in Zambia occurs in this age group across the country [49]. In this study, frequency of febrile malaria was highest (31.8%) in the 0–4 years age group and lowest (23.3%) in the 15 years and above group. There was no significant difference in parasitaemia in febrile patients of the three age categories (*P* > 0.05) suggesting a nonimmune population and an area of low transmission. The above 10% parasitaemia observed in children under 5 years of age confirmed that malaria had again become endemic in peri-urban Lusaka [18]. 

The knowledge and attitudes survey indicated the need for intensified information, education and communication (IEC) on malaria and its prevention. The 46% knowledge level on vector control interventions indicated a weakness in individual efforts to prevent the disease. Population expansion and its health impact has been epitomized by sub-Saharan Africa. In many malaria endemic countries, including Zambia, the population has doubled in the past two decades, thus greatly increasing the absolute numbers of those at risk [53]. Accordingly, the peri-urban settlements experience the highest population growth rates [11]. This was demonstrated in peri-urban Lusaka where family demographic data showed an average of seven residents with at least one child less than five years per household. Thus, suggesting that congestion in households was probably one of the factors contributing to the increased transmission of malaria in these settings.

It has equally been established that human migration contributes markedly to malaria transmission [54]. In areas of endemicity, encroaching transmission has been demonstrated in areas previously free of transmission and local transmission has been conclusively demonstrated in many African cities [55, 56]. These findings are corroborated in this study which confirmed local transmission in Lusaka as 80% subjects with definitively diagnosed malaria had no history of travel. It was established that there is no significant contribution of migration towards malaria transmission in peri-urban Lusaka (*P* > 0.05). Local transmission of malaria was further strongly inferred by high parasitaemia in children under the age of five and the presence of gametocyte bearers and efficient vectors in the community that perpetuated the transmission cycle. Congestion in households together with the appreciably low levels of knowledge on control and prevention compounded the situation. 

The pragmatic data reported on here was an essential prerequisite of evidence-based and effective vector control efforts. The high malaria infection rates in peri-urban Lusaka could be ascribed to the definitively demonstrated local transmission. This necessitated the institution of appropriate control strategies based on the prevailing transmission paradigm. The presence of *An. gambiae* complex species and characterization of their breeding attributes required an integrated vector management (IVM) approach to effectively control transmission. It is noteworthy, that this preintervention study had limitations as the surveys were conducted during the dry season which markedly influenced the malaria vector and parasite populations.

Clearly, the malaria epidemiology in peri-urban Lusaka required an integrated approach involving IRS and ITNs against the adults and larval source management (LSM) against the aquatic stages. Information education and communication (IEC) to increase awareness and knowledge about malaria vector control needed to be intensified. Following this study, IVM was introduced in Lusaka with IRS and ITNs as main thrust interventions and IEC has been strengthened [57]. This has reduced malaria parasite rates to appreciably minimal levels (<1%) [58]. To clear the residual transmission, LSM is being implemented in Lusaka. While monitoring and evaluation of vector control interventions has been strengthened [45], it is imperative that a comprehensive entomological and epidemiological surveillance system is established to detect any increase in the malaria case load. 

## Figures and Tables

**Table 1:** Entomological survey data.

Breeding sites, larval densities and ratios				
Habitat type	Transient	Semipermanent	Permanent	Total
Abundance	6 (43%)	5 (36%)	3 (21%)	14
Larvae collected	1213 (66%)	508 (28%)	119 (6%)	1840
Anophelenes	212 (17.5%)	161 (31.7%)	30 (25%)	403
Culicines	1001 (82.5%)	347 (68.3%)	89 (75%)	1437
An-Cul ratio	0.21	0.46	0.34	
Larvae/250 mL	70	45	20	

Vector molecular identification				
	Kalikiliki		Chazanga	

*An. gambiae *ss.	11 (58%)		7 (37%)	
*An. arabiensis*	0		1 (5%)	

**Table 2:** Parasitological survey data.

Parasitemia in febrile patients by age and sex				
Age group	0–4 yrs	5–15 yrs	>15 yrs	Total
Number surveyed	52*	14*	63*	129
	38^†^	21^†^	109^†^	168
Frequency (age)	27 (30%)	9 (25.7%)	40 (23.3%)	76
Frequency (sex)	15*	4*	26*	45
	12^†^	5^†^	14^†^	31

Parasite densities by age				

1–10/100 O.I	25 (62.5%)	3 (33.3%)	15 (55.6%)	43
11–100/100 O.I	9 (19.0%)	2 (22.2%)	4 (17.4%)	15
10/O.I	3 (7.5%)	1 (11.1%)	3 (7.5%)	7
>10/O.I	3 (7.5%)	1 (11.1%)	5 (18.5%)	11

O.I: oil immersion field, ^†^female, and *male.
